# The Effects of New Design of Access Hole on Porcelain Fracture Resistance of Implant-Supported Crowns

**Published:** 2015-03

**Authors:** Reza Derafshi, Mitra Farzin, Masoumeh Taghva, Hossein Heidary, Berivan Atashkar

**Affiliations:** 1 Dept. of Prosthodontics, School of Dentistry, Shiraz University of Medical Sciences, Shiraz, Iran;; 2 Postgraduate Student of Prosthodontics, Dept. of Prosthodontics, School of Dentistry, Shiraz University of Medical Sciences, Shiraz, Iran;

**Keywords:** Dental Implants, Cement-retained, Fracture Resistance, Metal Ceramic Crowns

## Abstract

**Statement of the Problem:**

One disadvantage of cement-retained crowns is the lack of predictable irretrievability. This problem can be overcome through designing a screw access hole in the metal substructure of cement-retained restoration and using porcelain stain to define this area.

**Purpose:**

This study aimed to evaluate the influence of existence of screw access hole on porcelain fracture resistance of metal-ceramic implant-supported crowns.

**Materials and Method:**

Thirty six standardized metal-ceramic crowns were fabricated and divided into 3 groups (n=12); group 1 conventional cement-retained metal-ceramic crowns as control group, group 2 cement-retained MC crowns in which porcelain stain was used to define the location of screw access channel, and group 3 cement-retained metal-ceramic crowns in the metal substructure of which a hole and ledge was designed in the location of screw access channel. The specimens were cemented (TempBond, Kerr) to their dedicated abutments. A hole was made in the location of screw access channel in group 2 and 3 and filled with photo-polymerized composite resin (3M; ESPE). All specimens were thermocycled and loaded in universal testing machine at crosshead speed of 2mm/min until fracture. Mean values of load at fracture were calculated in each group and compared with One-way ANOVA (α=0.05).

**Results:**

Mean value of the load required to fracture the restorations was 1947±487 N in group 1, 1927±539 N in group 2, and 2170±738 N in group 3. No statistically significant difference was found between the fracture resistance values of the three groups (*p*> 0.05)

**Conclusion:**

Presence of screw access channel in cement-retained implant restorations does not compromise fracture resistance.

## Introduction

Dental implants have been approved for their durable predictable success in treatment of both completely[1-2] and partially edentulous patients.[3-4] Metal ceramic restorations are generally used during the restoration phase of implant, particularly in treatment of partially edentulous cases.[5] Implant-supported crowns can be either screw-retained[6-7] or cement-retained;[8-9] however, there still exists controversies over the best retention type for implant-supported restorations.[10-14]

Retrievability is the main advantage of screw-retained prosthesis,[15-16] because the prosthesis might need to be removed to repair the crown (in case of ceramic-fracture or screw loosening). This feature also provides a better assessment of oral hygiene and peri-implant probing, as well as replacing the components in case of screw loosening or fracture.[16-18] However, the laboratory procedures required for screw-retained restorations are usually more sophisticated, expensive, and associated with inherent mechanical complications such as screw-loosening and fractures.[19-20] It is generally difficult and costly to remove and replace the fractured screws.[15]

Furthermore, natural occlusal morphology might be interfered due to the presence of a screw access opening,[21] which might also disrupt the porcelain continuity and lead to unstable occlusal contents.[22-23]As reported by a number of studies, presence of screw access opening in these restorations reduces the fracture resistance of the porcelain.[15, 22, 24-25]

Among the advantages of cement-retained restorations are the lower costs of fabrication, facilitated procedure of implant restoratives, and a better passive fit, as well as preventing the interference of screw access opening with the esthetic or the occlusion of the restoration.[8, 25] Meanwhile, cement-retained restorations have some drawbacks including the difficulty in retrieving and removing the excess cement around the crown, in addition to cement loss which may lead to peri-implant inflammation.[15, 26-27]

Cement-retained restorations have been advised for treating partial edentulous patients with implant[16] and they are better to be the first treatment option when esthetic is concerned.[28] Besides, cement-retained implant prosthesis is chiefly concerned with the difficulty of being removed when the abutment screw has loosened or the porcelain has fractured and need to be corrected.

Literatures have suggested several methods to provide retrievability of cement-retained implant restorations; using provisional cement is one of them, although the retention may be damaged.[28] Placing a lingual retrieval slot at the abutment/prosthesis interface is another solution.[29] Other approaches are making use of ceramic stain on the occlusal surface of posterior restoration,[15] digital photographs or vacuum-formed templates to identify the position of screw.[30] In these methods, the screw channel is filled with composite resin when the restoration is removed. One concern is that, existence of screw channel in the framework may jeopardize the strength of restoration.

In the present study, a special feature was designed in the metal framework to support the remaining porcelain after the screw access channel was created. There is little data in the literature regarding the fracture resistance of cemented prostheses with screw access channel. The current investigation aimed to evaluate the influence of access hole in the occlusal surface of cement-retained implant restoration with and without specially designed feature on the fracture resistance of the restoration. The null hypothesis was that creating screw access on cement-retained crowns to permit retrievability would not compromise the strength of restoration. 

## Materials and Method

**Figure 1:**
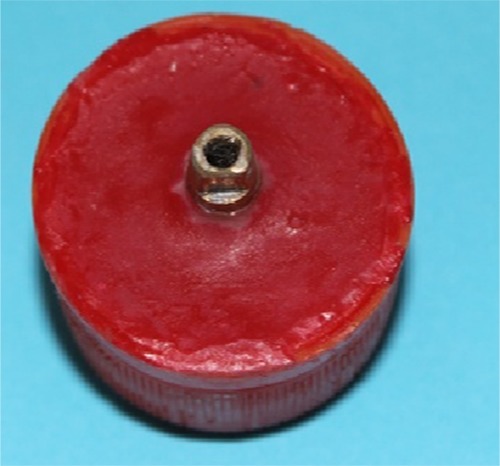
Brass die embedded in acrylic resin block

A 5×6.5 implant analog (Dio Corp.; Busan, Korea) was connected to a straight abutment. Then it was duplicated as 36 brass dies by using a lathe (CNC 350; Arix Co., Tainan Hesin, Taiwan). Then, five millimeters of each die was embedded vertically in an acrylic resin block with the aid of a dental surveyor (Figure 1).

**Figure 2:**
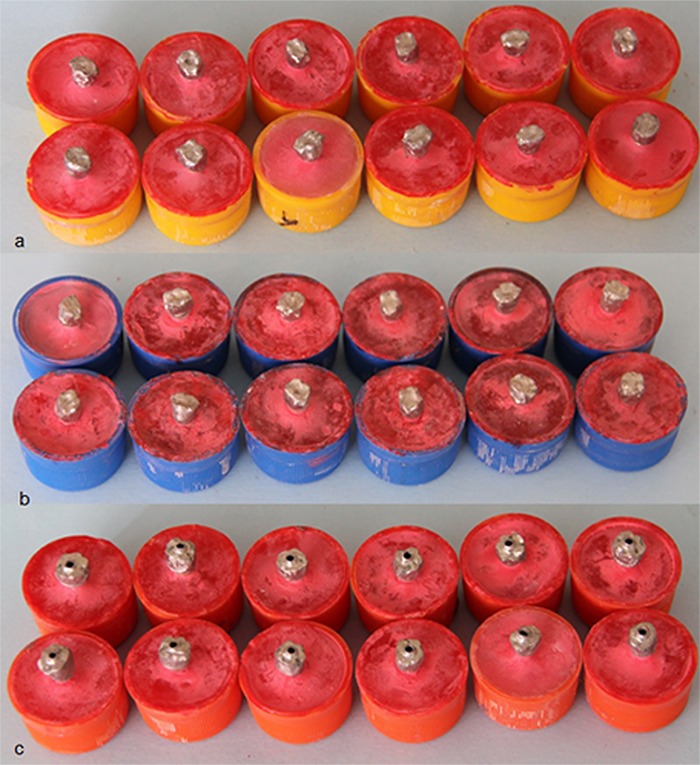
: 12 conventional metal-ceramic implant frameworks of group 1  b: Group 2; implant framewoks  c: Group 3;  implant frameworks

**Figure 3:**
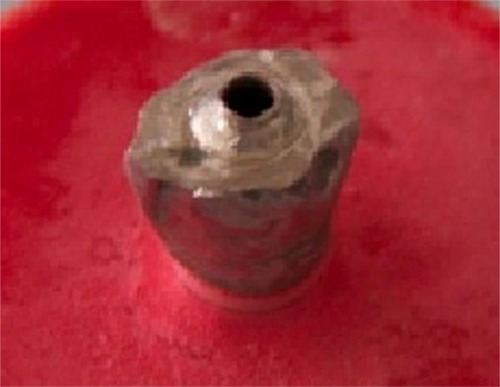
The ledge designed in the location of screw access channel of the third group of metal ceramic crowns

Subsequently 36 metal ceramic crowns were fabricated with 3 designs; group 1 consisted of 12 conventional metal-ceramic crowns with cement-retained restorations as control group (Figure 2a), group 2 included 12 crowns with porcelain stain used to define the location of screw access channel (Figure 2b). Group 3 was constituted of 12 crowns during framework fabrication of which, a ledge was designed in wax-up in the location of screw access channel (Figure 2c). The ledge was 1.5 mm in height and 1 mm in thickness around a 2-mm hole in the center of occlusal surface (Figure 3). This area was defined in the crown by using porcelain stain.

Porcelain application to all specimens was standardized by using a silicone index. The crowns were then cemented on their dedicated master dies with zinc oxide-eugenol cement (TempBond; Kerr Mfg Co., Romulus, MI) in rocking motion and held in place with constant finger pressure until the cement set. The excess cement was removed using an explorer. A hole was made in the location of screw access channel in group 2 and 3 by using a 2-mm fissure bur on a high speed handpiece, and was filled with photo-polymerized composite resin (3M; ESPE Dental Products, Canada).

**Figure 4:**
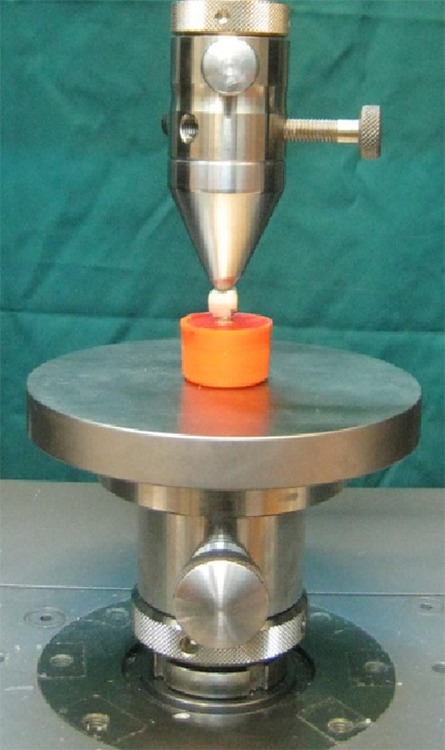
Frontal view of specimen positioned in the universal testing machine

Finally, all specimens were thermocycled for 500 cycles between 50-65^°^C for 30 seconds with 12-second intervals according to the literature.[31-32] Each specimen was subjected to vertical compression load by using a universal testing machine (Zwick-Roell Z020; Zwick Gmb H & Co. KG, Ulm, Germany) (Figure 4).

**Figure 5:**
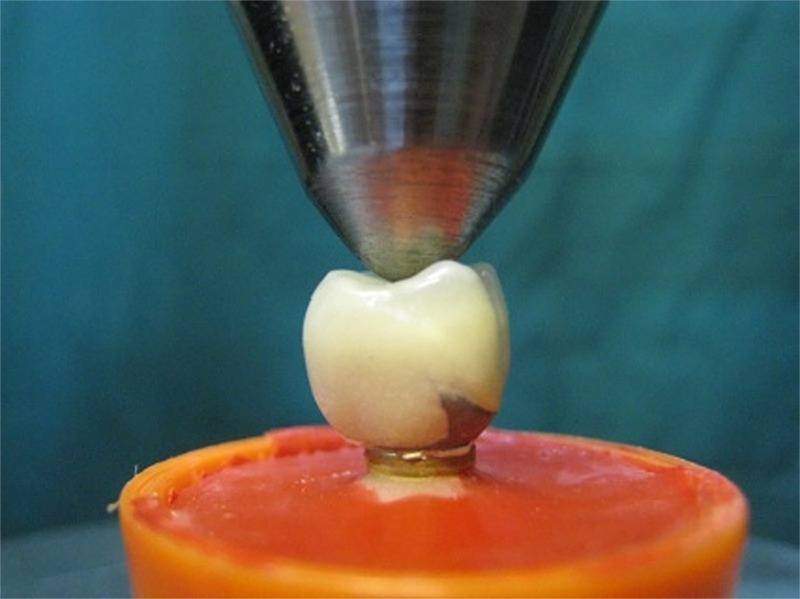
Close up view of loading apparatus

The force was applied perpendicular to the occlusal surface in the central part of the restoration with a cross-head speed of 2mm/min. In order to simulate the contact established by the opposing tooth, the rounded edges of the loading pin simultaneously contacted the triangular ridges of both buccal and lingual cusps of the crowns (Figure 5). The specimens were loaded to failure. Maximum values of loads at failure were recorded for each specimen. Mean values of fracture resistance for all groups were calculated and compared by using one-way ANOVA. The level of statistical significance was set at α=0.05.

**Table 1:** Fracture load (newton) of the three groups

	**N**	**Mean**	SD*	**Min**	**Max**
Group 1	12	1947	487	1370	3120
Group 2	12	1928	539	766	2610
control	12	2190	738	1210	3810

*Standard deviation

## Results

The mean fracture resistance value was 2190±738 N in group 3 (special design), 1928±539 N in group 2 and 1947±487 N in control group (Table 1). No statistically significant difference was detected among the three groups regarding the fracture resistance value (*p*= 0.491).

## Discussion

With respect to the obtained results of the present study, the null hypothesis cannot be rejected. The fracture resistance of specially designed cement-retained crowns with screw access was higher than the other two groups; this difference was not significant though (*p*> 0.05).

Zarone *et al.*[23] evaluated the fracture resistance of screw-retained versus cement-retained single metal-ceramic crowns. They found no significant difference between the two groups. However, fracture resistance values of cement-retained crowns were higher; which is in accordance with the results of the current study.

In another study, Karl *et al.*[32] observed more chipping fractures in screw-retained fixed dental prostheses than cemented models. They did not fill the access opening with any materials. The authors proposed filling the access hole with intraoral ceramic-repair resin composites to stabilize the occlusal surface of screw-retained implant restorations.

Several studies [22, 32-34] revealed the fracture resistance of screw-retained restorations with screw access hole to be less than cement retained restorations. The difference between the results obtained by the current study and other studies can be attributed to the cementing of the specimens. In the present study, the specimens were cement-retained with an access hole, while in other studies the specimens were screw-retained with an access hole which might had contained a screw or not. Other reasons that justify the difference in results could be related to the different procedures of fabricating the specimens, different sizes of screw access opening, filling the access hole with composite resin or leaving it unfilled, the type of resin repair system used, the type and cycles of force used for loading, the area on which the force was applied, luting the restorations or not, and the type of cement which was used. It should be noted that the minimum load which caused fracture in specimens in the aforementioned studies was more than the maximum masticatory forces.[35] Therefore, both types of the restorations can be considered predictable in the implant prosthetic restoration phase.

The especially cement-retained design proposed in the present study offered the advantages of cement-retained restorations in conjunction with the likelihood of the restoration retrievability. Furthermore, according to the study of Rocha *et al.*, screw access hole in cement-retained implant restoration had not any negative influence on the retention.[36]

There were some limitations in the present study, one of which was the single static force that was used to load the specimens and it differed from the dynamic load in the oral environment. In mouth, the restoration may also fracture due to fatigue loading.[37] In the present study, the crowns were cemented using zinc oxide-eugenol cement which is used more clinically and no permanent cement was tested.

Future researchers are recommended to investigate larger sample sizes under physiologic fatigue loading. Employing different types of cements for luting the specimens is also advised. 

## Conclusion

Within the limitations of this study, the following conclusions are drawn:

- Presence of screw access opening in cement-retained implant restoration would not compromise the fracture resistance.
- Designing a ledge in the framework of cement-retained implant restorations in the location of screw access opening could be a useful method to retrieve restoration, without compromising its strength.

## Notes

**Conflicting Interest: **The authors declare no conflict of interest.
